# Origin of Plants

**Published:** 1879-09

**Authors:** 


### ORIGIN OF PLANTS.

Cabbage grew wild in Siberia; buckwheat originated in Siberia; celery originated in Germany; the potato is a native of Peru; the onion originated in Egypt; tobacco is a native of South America; millet was first discovered in India; the nettle is a native of Europe; the citron is a native of Asia; oats originated in North Africa; rye came originally from Siberia; parsley was first discovered in Sardinia; the parsnip is a native of Arabia; the sunflower was brought from Peru; spinach was first cultivated in Arabia; the pear and apple are from Europe; the horse chestnut is a native of Thibet; the quince came from Island of Crete; the radish is a native of China and Japan; the pear is supposed to be of Egyptian origin; the horse radish came from the south of Europe.

